# Selling sex in the context of substance use: social and structural drivers of transactional sex among men who use opioids in Maryland

**DOI:** 10.1186/s12954-022-00697-3

**Published:** 2022-10-15

**Authors:** Joseph G. Rosen, Kristin E. Schneider, Sean T. Allen, Miles Morris, Glenna J. Urquhart, Saba Rouhani, Susan G. Sherman

**Affiliations:** 1 Department of International Health, Johns Hopkins Bloomberg School of Public Health, 615 N. Wolfe Street, E5031, Baltimore, MD 21205 USA; 2 Department of Health, Behavior and Society, Johns Hopkins Bloomberg School of Public Health, 624 N. Broadway, Baltimore, MD 21205 USA

**Keywords:** Male sex work, Injection drug use, Food insecurity, Sexuality, Opioid epidemic, USA

## Abstract

**Background:**

Transactional sex is an important driver of HIV risk among people who use drugs in the USA, but there is a dearth of research characterizing men’s selling and trading of sex in the context of opioid use. To identify contextually specific factors associated with selling or trading sex in a US population of men who use drugs, we cross-sectionally examined social and structural correlates of transactional sex among men who use opioids (MWUO) in Anne Arundel County and Baltimore City, Maryland.

**Methods:**

Between July 2018 and March 2020, we used targeted sampling to recruit men reporting past-month opioid use from 22 street-level urban and suburban recruitment zones. MWUO completed a 30-min self-administered interview eliciting substance use histories, experiences with hunger and homelessness, criminal justice interactions, and transactional sex involvement. We identified correlates of recent (past 3 months) transactional sex using multivariable log-binomial regression with cluster-robust standard errors.

**Results:**

Among 422 MWUO (mean age 47.3 years, 73.4% non-Hispanic Black, 94.5% heterosexual), the prevalence of recent transactional sex was 10.7%. In multivariable analysis, younger age (adjusted prevalence ratio [aPR] 0.98, 95% confidence interval [95% CI] 0.97–0.99, *p* < 0.001), identifying as gay/bisexual (aPR = 5.30, 95% CI 3.81–7.37, *p* < 0.001), past-month food insecurity (aPR = 1.77, 95% CI 1.05–3.00, *p* = 0.032), and injection drug use in the past 3 months (aPR = 1.75, 95% CI 1.02–3.01, *p* = 0.043) emerged as statistically significant independent correlates of transactional sex.

**Conclusions:**

Synergistic sources of social and structural marginalization—from sexuality to hunger, homelessness, and injection drug use—are associated with transactional sex in this predominantly Black, heterosexual-identifying sample of MWUO. Efforts to mitigate physical and psychological harms associated with transactional sex encounters should consider the racialized dimensions and socio-structural drivers of transactional sex among MWUO.

## Background

Harms related to transactional sex fall at the intersection of two co-occurring watersheds in the USA: the HIV epidemic and the opioid crisis. A global meta-analysis found that cisgender men who have sex with men (MSM) selling or trading sex exhibited 34% higher odds of HIV infection compared to MSM without transactional sex histories [1]. HIV burdens among people who sell/trade sex are elevated due, in part, to the conditions in which exchange sex occurs. For instance, transactional sex motivated by financial instability may disincentivize condom use or constrain condom negotiation capacity altogether [2, 3]. In the context of criminalized sex work and drug use, these same conditions also amplify the risk environment for condom coercion, violence, and acquisition of HIV and other sexually transmitted infections [4–8].

Furthermore, sharp increases in opioid use over the last decade, coinciding with the introduction of potent synthetic opioids like fentanyl and its analogs into drug markets [9], have exacerbated socio-structural vulnerabilities (e.g., unemployment, homelessness, income insecurity) that are closely associated with the selling and trading of sex [10–12]. Transactional sex and its socio-structural antecedents, therefore, are mutually reinforcing in the context of opioid use, whereby selling or trading sex exacerbates the very adversities that prompt people to exchange sex for money, drugs, or basic needs [13].

Scientific inquiry into the drivers of transactional sex among people who use drugs focuses almost exclusively on cisgender women, who relative to cisgender men encounter more gender-based inequities fueling transactional sex and fewer barriers to selling sex to a market dominated by male procurers [14]. Nevertheless, transactional sex among men is an important, albeit understudied, dimension of the US opioid crisis. Little is known about men’s transactional sex in the context of opioid use, as studies of transactional sex in men tend to focus exclusively on MSM [15–18], whose substance use patterns and associated vulnerabilities may be distinct from other men who use opioids (MWUO), both heterosexual and non-heterosexual. The available literature links transactional sex among MWUO to polysubstance use, injecting drugs, receptive syringe sharing, housing instability, and violence victimization [19–22]. Importantly, these insights are gleaned predominantly from studies conducted internationally and may, thus, have limited relevance to the US context.

To identify contextually specific factors associated with selling or trading sex in a US population of men who use drugs, we examine social and structural correlates of transactional sex among MWUO in urban and suburban settings in Maryland—a US state with high burdens of both opioid-involved overdose deaths and new HIV infections [23, 24].

## Methods

### Setting and design

We leveraged data from Peer Harm Reduction of Maryland Outreach Tiered Evaluation (PROMOTE), a multi-method study of people who use opioids in Anne Arundel County and Baltimore City, Maryland [25–28]. Baltimore City is a densely populated urban setting, with approximately 600,000 inhabitants [29]. Although formally incorporated into Baltimore City’s metropolitan statistical area, Anne Arundel County is predominantly suburban, with a constellation of small towns and the city of Annapolis (the state capital) [30]. Anne Arundel and Baltimore rank among the highest jurisdictions in Maryland for opioid-related deaths, reporting 251 and 1,028 fatal overdoses, respectively, in 2020 [24]. Baltimore City is among the most impacted municipalities of the US opioid crisis, with an age-adjusted overdose mortality rate of 96 deaths per 100,000 residents [24].

We identified 22 street-level recruitment zones (15 in Baltimore, 7 in Anne Arundel) using geolocated drug-related arrest and overdose data availed by city and county police precincts. We extracted time signatures associated with arrests to construct venue-day-time sampling units, which informed study recruitment activities across geographic areas. We collected data at two distinct time periods in Baltimore (July–October 2018, April–July 2019) and in a single wave in Anne Arundel (November 2019–March 2020). Participants were aged ≥ 18 years and reported non-medical opioid use (i.e., any non-prescribed opioid, including heroin, fentanyl, and painkillers not prescribed by a healthcare provider) in the past 6 months (Anne Arundel) or past month (Baltimore). Eligible individuals providing verbal informed consent completed a 30-min audio computer-assisted self-interview, administered on tablets in a study van. Participants were offered referrals to various health-related (e.g., syringe services programs, drug treatment) and social (e.g., housing, food assistance) services after completion of the survey. Study staff received training in mental health first response and trauma-informed interviewing. We compensated participants with a $25 preloaded Visa card.

### Measures

Our primary outcome was transactional sex, which we ascertained from responses (yes or no) to the question, “In the past 3 months, did you sell or exchange oral, vaginal, or anal sex for things like money, food, drugs, or a place to stay?”.

Demographic information collected from participants included age (in years); current relationship status (single, partnered but unmarried, married); and sexuality (heterosexual, gay, bisexual, queer, or other), which was dichotomized to compare men who identified as gay or bisexual to heterosexual men. Indicators of economic and structural vulnerability included completed education (completed secondary school/General Education Development (GED) test or less); any arrest in the past year (yes or no); current homelessness, based on self-report of whether participants consider themselves homeless (yes or no); food insecurity, defined as going to sleep hungry at least once weekly in the past month (yes or no); and current employment status (employed full-time/part-time or unemployed).

Recent (past 3 months) substance use variables included any injection drug use (yes or no); receptive injection equipment sharing, defined as using injection equipment previously used by someone else (yes or no); drug economy involvement, defined as making, selling, or trading any illicit drugs; typically using drugs in any semipublic or public spaces, including streets, parks, abandoned buildings, shooting galleries, cars, buses, metros, stairways, or public bathrooms (yes or no); solitary drug use, defined as primarily using drugs alone or in the presence of bystanders (yes or no); and any non-fatal drug overdoses (yes or no).

Lastly, measures of health and well-being included current health insurance status (coverage by a government-subsidized/public or private insurance provider or uninsured); history of mental health care provision (ever or never); self-reported Hepatitis C status (positive or negative); any emergency room visit in the past 3 months (yes or no); syringe service program (SSP) accessibility (ever accessed an SSP for any service or never); and ever taken pre-exposure prophylaxis (PrEP) for HIV prevention (yes or not).

### Analysis

We pooled data across Anne Arundel and Baltimore recruitment zones, restricting the analytic sample to cisgender men reporting opioid use in the past month (*N* = 422), excluding Wave 2 Baltimore participants (*n* = 11) who self-reported participating in the first survey wave. After calculating descriptive sample statistics in Stata/IC 15.1 (StataCorp LLC, College Station, TX), we implemented Fisher’s exact tests to identify statistically significant correlates of transactional sex. We included covariates associated with transactional sex at the *p* < 0.05 level in a multivariable log-binomial regression model, with standard errors clustered at the recruitment zone level to account for non-independent observations within geographic areas. A model-based variance inflation factor (VIF) score guided the removal of covariates (i.e., current homelessness) exhibiting considerable multicollinearity (VIF ≥ 2.5) [31]. We used model-wise deletion to address missing data (*n* = 10, ~ 2% of all observations) in multivariable analysis. We report regression coefficients as adjusted prevalence ratios (aPR) with 95% confidence intervals (95% CI).

## Results

Table 1 summarizes pooled sample characteristics for the 422 cisgender men with past-month opioid use in Anne Arundel County (*n* = 98, 23.2%) and Baltimore City (*n* = 324, 76.8%). The mean age was 43.7 years (*std. dev.* 11.4 years). Most participants identified as non-Hispanic Black (73.4%), single (72.5%), and heterosexual (94.5%). Economic and structural vulnerabilities were highly prevalent, including current homelessness (66.1%), food insecurity in the past month (58.3%), and current unemployment (85.7%). Over a third (38.7%) had less than secondary school-level education, and one-fourth (26.1%) reported being arrested in the past year. Over a third reported injecting drugs in the past 3 months (38.4%), of whom 21.6% receptively shared injection equipment. Over half reported making, selling, or trading drugs in the past 3 months (56.8%). Three-quarters of men reported any recent semipublic or public drug use (74.3%). Almost a quarter of men reported any solitary drug use in the past 3 months (22.3%), and 15.7% reported a recent drug overdose. In terms of health and well-being, most men had public or private insurance (78.8%) and ever received mental health care (64.4%). Over one-fourth reported their Hepatitis C status as positive (25.6%) and a recent emergency room visit (26.6%), respectively. Over a third of MWUO reported accessing an SSP (39.8%). Few, however, had ever taken PrEP (6.4%).

**Table 1:** Descriptive sample statistics among cisgender men who used opioids in the past month, stratified by transactional sex status—Anne Arundel County and Baltimore City, Maryland

Characteristics (*n*, %)	Overall *N* = 422	Transactional sex, past 3 months		Fisher’s exact *p* value
		*No* (*n* = 377, 89.3%)	*Yes* (*n* = 45, 10.7%)	
*Socio-demographics*				
Age, in years (*mean*, *std. dev.*)*	47.3 (11.4)	48.0 (11.2)	41.4 (11.9)	** < 0.001**
Race and ethnicity				0.215
Non-Hispanic Black	303 (73.4)	273 (74.0)	30 (68.2)	
Non-Hispanic White	67 (16.2)	61 (16.5)	6 (16.2)	
Hispanic and/or other	43 (10.4)	35 (9.5)	8 (18.2)	
Current relationship status				0.337
Single	306 (72.5)	273 (72.4)	33 (73.3)	
Partnered but unmarried	74 (17.5)	64 (17.0)	10 (22.0)	
Married	42 (10.0)	40 (10.6)	2 (4.4)	
Sexual orientation				** < 0.001**
Heterosexual/straight	397 (94.5)	362 (96.5)	35 (77.8)	
Gay or bisexual	23 (5.5)	13 (3.5)	10 (22.2)	
Locality				0.939
Anne Arundel	98 (23.2)	88 (23.3)	10 (22.2)	
Baltimore (2018)	167 (39.6)	148 (39.3)	19 (42.2)	
Baltimore (2019)	157 (37.2)	141 (37.4)	18 (35.6)	
*Economic and structural vulnerabilities*				
Completed education				0.747
Secondary school/GED or higher	258 (61.3)	229 (60.9)	29 (64.4)	
Less than secondary school/GED	163 (38.7)	147 (39.1)	16 (35.6)	
Arrested, past 12 months				**0.002**
No	312 (73.9)	288 (76.4)	24 (53.3)	
Yes	110 (26.1)	89 (23.6)	21 (46.7)	
Currently homeless				**0.019**
No	143 (33.9)	135 (35.8)	8 (17.8)	
Yes	279 (66.1)	242 (64.2)	37 (82.2)	
Food insecurity, past month				**0.006**
Went to sleep hungry < 1 × weekly	176 (41.7)	166 (44.0)	10 (22.2)	
Went to sleep hungry ≥ 1 × weekly	246 (58.3)	211 (56.0)	35 (77.8)	
Current employment status				0.655
Full-time or part-time employment	60 (14.3)	55 (14.6)	5 (11.1)	
Unemployed	361 (85.7)	321 (85.4)	40 (88.9)	
*Substance use, past 3 months*				
Injection drug use				**0.035**
None	260 (61.6)	239 (63.4)	21 (46.7)	
Any	162 (38.4)	138 (36.6)	24 (53.3)	
Receptive syringe sharing**				**0.015**
None	127 (78.4)	113 (81.9)	14 (58.3)	
Any	35 (21.6)	25 (18.1)	10 (41.7)	
Made, sold, or traded drugs				**0.025**
No	180 (43.2)	168 (45.0)	12 (27.3)	
Yes	237 (56.8)	205 (55.0)	32 (72.7)	
Drug use setting				0.717
Private use only	103 (25.7)	93 (26.1)	10 (22.7)	
Any semipublic or public use	298 (74.3)	264 (74.0)	34 (77.3)	
Solitary drug use				0.850
Used in the presence of others	327 (77.7)	291 (77.4)	36 (80.0)	
Used alone	94 (22.3)	85 (22.6)	9 (20.0)	
Non-fatal drug overdose				0.182
None	348 (84.3)	315 (85.1)	33 (76.7)	
Any	65 (15.7)	55 (14.9)	10 (23.3)	
*Health and well-being*				
Current health insurance status				0.845
Public or privately insured	331 (78.8)	297 (79.0)	34 (77.3)	
Uninsured	89 (21.2)	79 (21.0)	10 (22.7)	
Mental health service provision				0.102
Never received mental health care	149 (35.6)	138 (37.0)	11 (24.4)	
Ever received mental health care	269 (64.4)	235 (63.0)	34 (75.6)	
Hepatitis C status				0.365
Negative	313 (74.7)	282 (75.4)	31 (68.9)	
Positive	106 (25.6)	92 (24.6)	14 (31.1)	
Emergency room visit, past 3 months				0.478
No	309 (73.4)	278 (73.9)	31 (68.9)	
Yes	112 (26.6)	98 (26.1)	14 (31.1)	
Accessed a syringe services program				0.502
No	254 (60.2)	229 (60.7)	25 (55.6)	
Yes	168 (39.8)	148 (39.3)	20 (44.4)	
Ever taken PrEP				0.057
No	392 (93.6)	353 (94.4)	39 (86.7)	
Yes	27 (6.4)	21 (5.6)	6 (13.3)	

Bolded values indicate statistically significant (*p* < 0.05) differences

*Covariates compared using Student’s *t* test. **Restricted to persons reporting injection drug use in the past 3 months (*n* = 162)

Across study sites, 10.7% of men reported recent transactional sex. MWUO who were younger (mean 41.4 vs. 48.0 years, *p* < 0.001), gay or bisexual (22.2% vs. 3.5%, *p* < 0.001), arrested in the past year (46.7% vs. 23.6%, *p* = 0.002), currently experiencing homelessness (82.2% vs. 64.2%, *p* = 0.019), and reported past-month food insecurity (77.8% vs. 56.0%, *p* = 0.006) were significantly more likely to report selling or trading sex in bivariate analyses. Other significant correlates of transactional sex included injection drug use (53.3% vs. 36.6%, *p* = 0.035), receptive syringe sharing (41.7% vs. 18.1%, *p* = 0.015), and drug economy involvement (72.7% vs. 55.0%, *p* = 0.025) in the past 3 months. While MWUO reporting transactional sex were over twice as likely to endorse lifetime PrEP use, this difference was only marginally significant (13.3% vs. 5.6%, *p* = 0.057).

In multivariable analysis (Table 2), younger age (aPR = 0.98, 95% CI 0.97–0.99, *p* < 0.001), identifying as gay or bisexual (aPR = 5.30, 95% CI 3.81–7.37, *p* < 0.001), food insecurity (aPR = 1.77, 95% CI 1.05–3.00, *p* = 0.032), and injection drug use (aPR = 1.75, 95% CI 1.02–3.01, *p* = 0.043) remained significantly associated with transactional sex.

**Table 2:** Unadjusted and adjusted prevalence ratios (aPR) and 95% confidence intervals (95%CI) of recent transactional sex obtained from log-binomial regression (*N* = 412)

Covariates	PR (95%CI)	*p* value	aPR (95%CI)	*p* value
Age, in years	**0.96 (0.94–0.98)**	**0.001**	**0.98 (0.97–0.99)**	** < 0.001**
*Sexual orientation*				
Heterosexual/straight	1.00	*Ref*	1.00	*Ref*
Gay or bisexual	**6.43 (4.52–9.15)**	** < 0.001**	**5.30 (3.81–7.37)**	** < 0.001**
*Arrested, past 12 months*				
No	1.00	*Ref*	1.00	*Ref*
Yes	**2.48 (1.50–4.12)**	** < 0.001**	1.49 (0.87–2.56)	0.151
Currently homeless*				
No	1.00	*Ref*		
Yes	**2.37 (1.07–5.23)**	**0.032**		
*Food insecurity, past month*				
Went to sleep hungry < 1 × weekly	1.00	*Ref*	1.00	*Ref*
Went to sleep hungry ≥ 1 × weekly	**2.50 (1.45–4.33)**	**0.001**	**1.77 (1.05–3.00)**	**0.032**
*Made, sold, or traded drugs, past 3 months*				
No	1.00	*Ref*	1.00	*Ref*
Yes	2.03 (0.91–4.52)	0.085	1.51 (0.70–3.27)	0.296
*Injection drug use, past 3 months*				
None	1.00	*Ref*	1.00	*Ref*
Any	**1.83 (1.02–3.31)**	**0.044**	**1.75 (1.02–3.01)**	**0.043**

Multivariable log-binomial model adjusted for all covariates presented in the table. Cluster-robust standard errors were implemented at the recruitment zone level

Bolded values indicate statistically significant (*p* < 0.05) differences

*Covariate excluded from multivariable analysis due to considerable multicollinearity (VIF ≥ 2.5) with food insecurity

## Discussion

Our study is among the first to examine social and structural drivers of transactional sex among MWUO in the USA We found that younger age, gay/bisexual identity, and overlapping sources of marginalization were associated with transactional sex in our sample of cisgender MWUO in urban and suburban Maryland. Food insecurity and injection drug use, specifically, were independently associated with transactional sex, although homelessness and arrest history emerged as significant bivariate correlates of selling or trading sex. Age was also inversely associated with transactional sex—a possible reflection of heightened marginalization among younger MWUO in our sample and/or increased market demands for sex with younger men [32, 33]. Taken together, our findings help identify social and structural drivers of MWUO’s transactional sex involvement as well as opportunities to mitigate harms associated with selling sex in the context of opioid use.

While the relationship between socio-structural vulnerabilities and transactional sex is well characterized in women [34, 35], less is known about the marginalization processes underpinning men’s transactional sex involvement, especially in the context of opioid use. We identified food insecurity as a significant independent correlate of transactional sex, which is well documented among women who use drugs but not men [12, 36, 37]. MWUO who injected drugs were also significantly more likely than MWUO who did not inject drugs to report transactional sex. Given that injection drug use has been linked to elevated disenfranchisement (e.g., unstable housing, violence, trauma, financial insecurity), injecting drugs could perpetuate the exchange of sex for money, drugs, or basic needs [38–41].

These findings should also be couched in the high rates of unemployment and criminal legal interactions observed in the study population. MWUO with past-year arrests were twice as likely to endorse transactional sex involvement. Arrest histories may elevate transactional sex propensities among MWUO who experience barriers to other employment or income-generating prospects, which may be attributed in part to prior convictions or pending criminal litigation [42, 43]. The near-universal levels of unemployment reported in the study population further reinforce the economic disenfranchisement experienced by the study population. Given the criminalization of drug possession and racialized dimensions of criminal legal encounters in the USA, it is unsurprising that transactional sex coincided with arrest histories and other manifestations of economic and structural marginalization in this predominantly Black-identifying population of MWUO [44, 45].

Sexuality emerged as another strong correlate of selling or trading sex in the context of opioid use. MWUO identifying as gay or bisexual were significantly more like to report transactional sex compared to their heterosexual-identifying counterparts. Because the procurers of transactional sex are overwhelmingly cisgender men, gay and bisexual MWUO may be more willing than heterosexual MWUO to sell sex because these transactional sex partnerships are likely concordant with their sexual orientations [20–22]. Elevated transactional sex involvement among gay and bisexual MWUO could also reflect underlying histories of social exclusion and isolation attributed to the sexual identities of gay and bisexual men, particularly among Black men. Studies have linked perceived homophobia and racialized disconnection from broader queer networks to increased sexual risk-taking, including condomless and transactional sex, among Black MSM in the USA [43, 46, 47]. These findings suggest that transactional sex among gay/bisexual MWUO in our sample is both a reflection of concomitant sources of marginalization (i.e., food insecurity, homelessness, injection drug use) and a potential consequence of racialized homophobia underpinning these very indicators of marginalization.

Our findings should be considered with several limitations in mind. First, data were self-reported and, therefore, subject to recall and responses biases. Second, unlike other studies [20, 48], we did not measure the genders of MWUO’s sexual partners, including those who paid for sex with participants, which could induce potential misclassification of sexuality among MWUO with discordant sexual identities and behaviors. Third, the absence of specific substance use indicators (e.g., types of drugs used and routes of administration, polysubstance use) and risk indicators (e.g., condomless sex, HIV status, physical and sexual violence victimization), which other studies have linked to transactional sex among men who use drugs [19, 20, 39], renders our findings susceptible to residual confounding. Fourth, our study’s focus on social and structural drivers of transactional sex overemphasizes measures that reflect deficits in the study population and underemphasizes asset-based measures (e.g., agency, resilience), which were not uniformly captured across survey waves. Future studies should take a strengths-based approach to examine correlates of transactional sex among MWUO. Fifth, the study’s moderate sample size likely attenuated statistical power to detect significant correlates of transactional sex, especially in multivariable analysis. Sixth, due to the study’s cross-sectional design, we cannot infer temporality from observed covariate associations with transactional sex. Lastly, our study population included primarily Black-identifying MWUO in urban and suburban Maryland, potentially limiting the transportability of our findings to rural MWUO or settings with distinct racial compositions.

## Conclusions

Our findings contribute to the scant literature on men’s selling and trading of sex in the context of opioid use. We demonstrate that synergistic sources of marginalization—from sexuality to hunger, homelessness, and injection drug use—are associated with MWUO’s transactional sex involvement. Efforts to mitigate physical and psychological harms associated with transactional sex encounters (i.e., diminished agency to negotiate safer sex, condom coercion, sexual violence, trauma) should consider the socio-structural drivers of transactional sex among MWUO. Integrating mental health care, legal support services, and HIV prevention (including PrEP provision) into frontline harm reduction programs like SSPs, which were relatively well accessed by participants, are potential vehicles for responding to adversities experienced by transactional sex-involved people who use drugs [49, 50]. Drug decriminalization offers additional opportunities for addressing concomitant sources of marginalization (i.e., arrest histories) experienced by MWUO, which were closely associated with transactional sex in our study population. Future research should interrogate the contribution of other adversities, including violence victimization and psychological distress, to men’s transactional sex behaviors, as well as harm reduction strategies that improve the health and well-being of transactional sex-involved MWUO.

## Data Availability

The data used in the present manuscript are available from the senior author (SGS) upon reasonable request.

## Abbreviations

GED: General Education Development
MSM: Men who have sex with men
MWUO: Men who use opioids
PrEP: Pre-exposure prophylaxis
SSP: Syringe services program
VIF: Variance inflation factor
